# Neutrophils in Inflammatory Bone Diseases

**DOI:** 10.1007/s11914-024-00865-3

**Published:** 2024-02-29

**Authors:** Carmelo Carmona-Rivera, Mariana J. Kaplan, Liam J. O’Neil

**Affiliations:** 1 Systemic Autoimmunity Branch, National Institute of Arthritis and Musculoskeletal and Skin Diseases, National Institutes of Health, Bethesda, MD 20892 USA; 2 Manitoba Centre for Proteomics and Systems Biology, Department of Internal Medicine, University of Manitoba, Winnipeg, MB Canada

**Keywords:** Neutrophils, Osteoclast, Citrullination, Carbamylation, NETs, Histones, Rheumatoid arthritis, Periodontitis

## Abstract

**Purpose of Review:**

In this review, we summarize the current evidence that suggests that neutrophils play a key role in facilitating damage to local bone structures.

**Recent Findings:**

Neutrophil infiltration is a hallmark of inflammatory bone diseases such as rheumatoid arthritis (RA) and periodontitis disease (PD). Both of these human diseases are marked by an imbalance in bone homeostasis, favoring the degradation of local bone which ultimately leads to erosions. Osteoclasts, a multinucleated resident bone cell, are responsible for facilitating the turnover of bone and the bone damage observed in these diseases. The involvement of neutrophils and neutrophil extracellular trap formation have recently been implicated in exacerbating osteoclast function through direct and indirect mechanisms. We highlight a recent finding that NET proteins such as histones and elastase can generate non-canonical, inflammatory osteoclasts, and this process is mediated by post-translational modifications such as citrullination and carbamylation, both of which act as autoantigens in RA.

**Summary:**

It appears that NETs, autoantibodies, modified proteins, cytokines, and osteoclasts all ultimately contribute to local and permanent bone damage in RA and PD. However, more studies are needed to fully understand the role of neutrophils in inflammatory bone diseases.

## Introduction

Inflammatory bone diseases such as rheumatoid arthritis (RA) and periodontitis disease (PD) are characterized by progressive bone destruction accompanied by chronic inflammation and infiltration of immune cells [1, 2]. While the precise etiology of these conditions is still unknown, it is considered that RA and PD develop as a result of genetic and environmental factors [3–5]. Dysregulation of inflammatory and immune processes in the synovium and gingiva in RA and PD respectively, accompanied by bone loss, highlight the role of immune cells in osseous health and integrity in these diseases. During the early stages of these conditions, neutrophilic infiltration into the affected tissues is one of the major hallmarks, and for many years, neutrophils have been implicated in bone destruction [6]. Neutrophils are sentinel cells and the most abundant white blood cells in circulation. These cells are endowed with powerful enzymes and anti-microbial peptides to protect the host from infections and other noxious stimuli through different mechanisms. One of these anti-microbial strategies is the formation of neutrophil extracellular traps (NETs). NETs are web-like structures of chromatin decorated with granule proteins and enzymes [7]. Dysregulation of NET formation has been linked to inflammation, autoimmunity, and end-organ damage [8•]. During NET formation, proteins are modified, and these modifications have been linked to aberrant immune responses in RA and PD [9, 10, 11•].

## Bone erosion in RA and PD

Bone remodeling is controlled by the coordinated activity of bone cells, osteoblasts (bone-forming), and osteoclasts (bone-resorbing). Imbalance of this process, tipping in favor of osteoclasts, leads to pathogenic bone erosion seen in multiple conditions [12–14]. Osteoclasts are specialized multinucleated cells derived from the myeloid compartment and﻿﻿ myeloid compartment and play a dominant role in bone resorption. Osteoclast formation is controlled by the master regulator receptor activator of nuclear factor-kB ligand (RANKL) and macrophage colony-stimulating factor-1 (M-CSF) [15]. Immune dysregulation leads to bone destruction in diseases such as RA and PD.

**Fig. 1:**
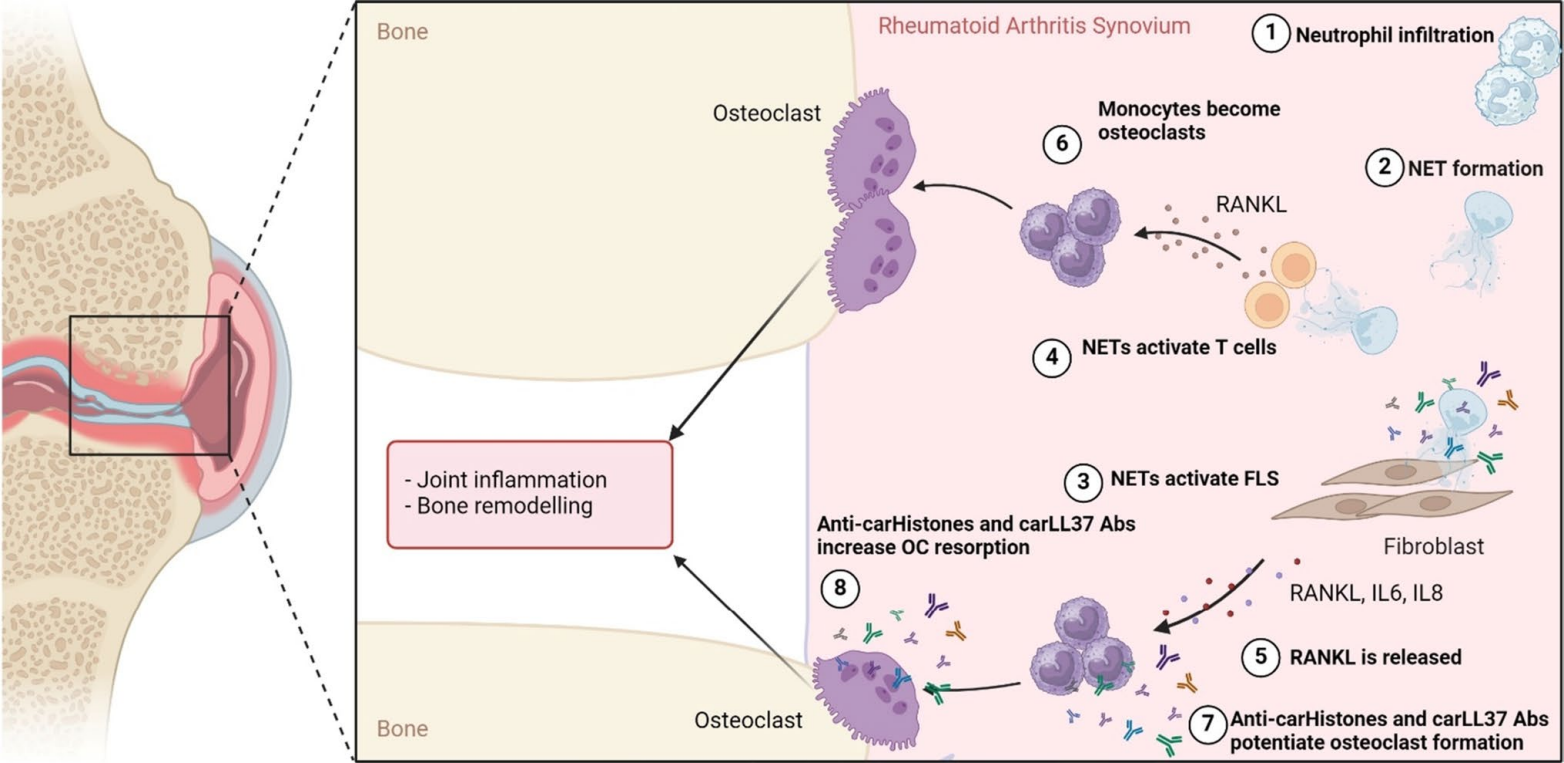
Role of neutrophils in bone erosion in rheumatoid arthritis (RA). (1, 2) Infiltrating neutrophils in RA synovium are primed by cytokines and autoantibodies, leading to enhanced capacity to form NETs (3, 4). NETs contain modified proteins that can serve as autoantigens in RA patients and activate fibroblast-like synoviocytes (FLS) and T cells. (5, 6) This activation leads to the release of receptor activator of nuclear factor kappa beta ligand (RANKL) and other pro-inflammatory cytokines that will activate monocytes. (7) Along with immune complexes of modified histones and LL37, monocytes can become osteoclasts. (8) These immune complexes also potentiate osteoclast’s resorption capacity leading to increased bone erosion. Diagram generated using BioRender.com

**Fig. 2:**
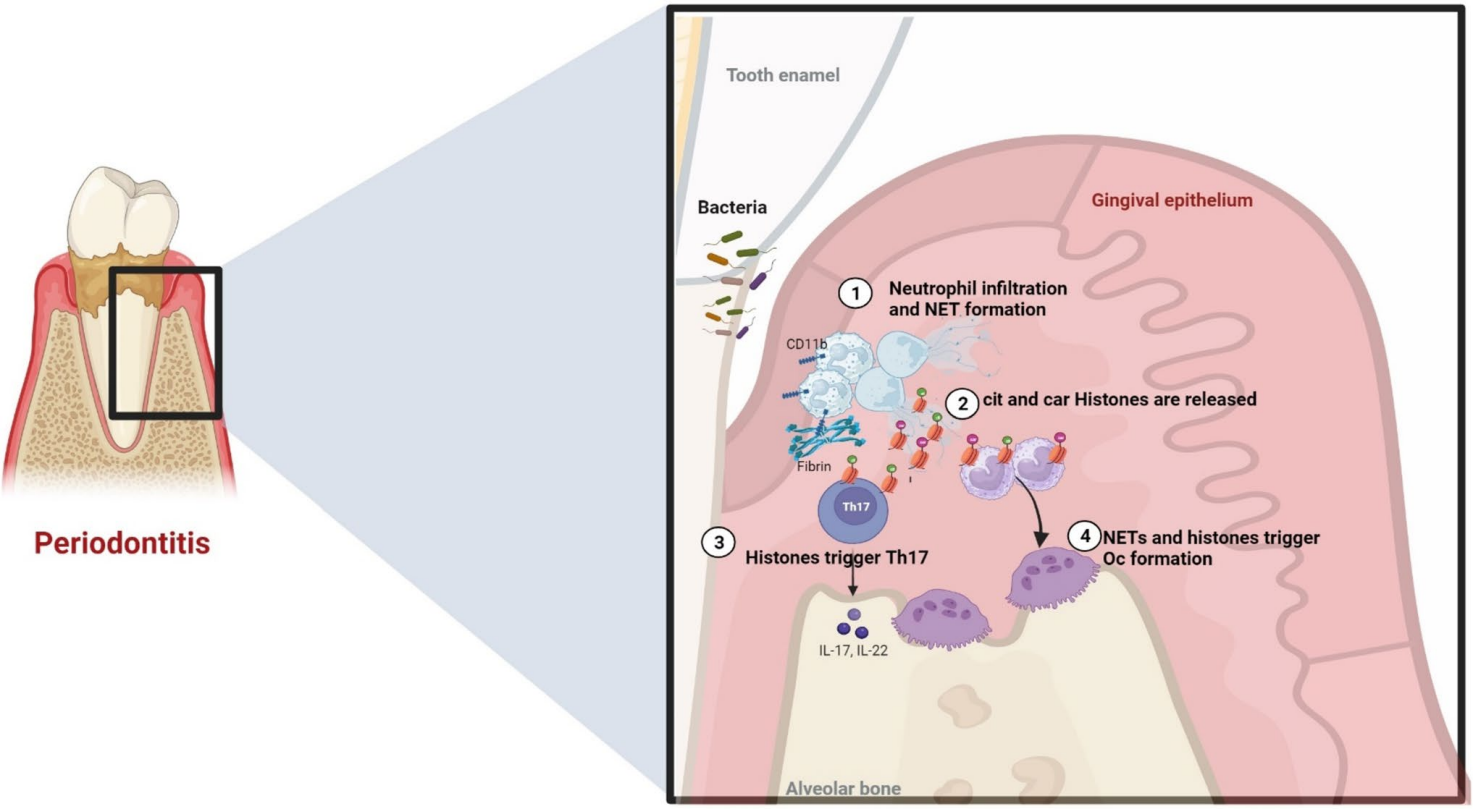
Role of neutrophils in bone erosion in periodontitis. (1) Bacteria and biofilm trigger inflammation of gingival tissue promoting recruitment of neutrophils. Bacteria, pro-inflammatory cytokines, and fibrin accumulation lead to NET formation and (2) the release of modified histones and enzymes such as neutrophil elastase. (3) Histone H3 is involved in the polarization of Th17 in periodontitis, (4) while histones and their modified forms lead to the formation of osteoclasts and increase bone loss. Diagram generated using BioRender.com

- *Rheumatoid arthritis (RA)* is a chronic systemic autoimmune condition characterized by inflamed synovial tissue, cartilage damage, and bone erosion [16]. Patients with RA develop antibodies against citrullinated peptides called ACPAs. These antibodies have been associated with disease severity and can be detected years prior to the onset of the condition. The development of RA is linked to genetic risk, driven primarily by the presence of the shared epitope (SE), an HLA-DRB1 risk allele [17] (Fig. 1).
- *Periodontitis disease (PD*) is a host-mediated chronic inflammatory disease associated with dental dysbiosis, and it is characterized by the destruction of soft tissue and bone loss [18]. This inflammation tends to persist until the affected tooth is removed or the microbial biofilm is treated (typically surgically) (Fig. 2).

Although RA and PD are distinct diseases, they are inextricably linked, due to their overlapping pathogenesis and the common co-occurrence of both diseases. A large number of epidemiological studies have shown a link between RA and PD, with the strongest association between ACPA-positive RA and PD [19]. This association is also driven by genetic risk, with an important gene-environment interaction between RA and PD that is strongly associated with more severe RA [20]. Furthermore, treatment of PD in RA patients is associated with improved arthritis disease activity, suggesting common pathways in both diseases [21]. RA patients develop autoantibodies to autoantigens derived from microbes associated with PD, namely, *Porphyromonas gingivalis* (*P. gingivalis*) [22–24]. RA autoantibodies can also target *P. gingivalis* [25, 26]. Monoclonal IgG from gingival B cells cross-react with *P. gingivalis*-derived citrullinated peptides [27]. Recently, it has been reported that oral microbes can migrate into the bloodstream, and this may drive inflammatory monocytes and B-cell responses that lead to flares of RA [28].

Chronic inflammation is considered a primary trigger of bone damage. Pro-inflammatory cytokines such as TNF, IL-6, IL-8, and IL-1 have been associated with bone loss in RA and PD [29]. In mouse models, the recruitment of CCL2 + inflammatory monocytes and their differentiation into osteoclasts via RANKL seem to play a central role in bone loss [30]. Synovial and periodontal inflammatory macrophages are considered central in disease progression through the production of pro-inflammatory cytokines [31–33]. TNF stimulates the production of RANKL and IL-6, which promotes osteoclastogenesis and directly stimulates the differentiation of osteoclasts from monocytes in mice [34]. In vitro human RA studies and in vivo models of arthritis suggest that macrophages and fibroblast-like synoviocytes (FLS) produce IL-6, which in turn promotes the production of RANKL [11•, 35, 36]. Inflammatory macrophages in RA and PD also produce IL-1 that promotes osteoclast differentiation in combination with TNF [37, 38] and indirectly by stimulating the recruitment of osteoclast precursors through IL-8 and CCL2 [39–41]. Further, other studies have shown that IL-1 can increase osteoclast activity on its own [42]. IL-17 is derived from Th17 cells, a specialized subset of CD4 + T cells that play a prominent role in autoimmunity. IL-17 appears to mediate inflammatory bone destruction through the excessive recruitment of neutrophils and neutrophil-mediated immunopathology [43]. Indeed, inhibition of IL-17 led to decreased neutrophil granulopoiesis and chemokine leading to decreased neutrophil recruitment into the tissue in murine periodontitis [44–46]. IL-17 is found in the synovial fluid of RA patients, which has been shown in vitro to lead to the production of osteoclasts in the presence of osteoblasts, suggesting that IL-17 increases the expression of RANKL on osteoclast-supporting cells [47]. In vitro cell cultures suggest that Th17 cells express higher levels of RANKL compared to Th1 and Th2 cells, further implicating them as osteoclast-supporting cells [48]. IL-17 also appears to be critical for cortical but not trabecular bone loss in mouse models, uniquely positioning itself to influence bone surfaces such as at the articular joints [49].

## Osteoclast Formation and Function

Osteoclasts form through the fusion of their progenitor, pre-osteoclast cells which are CD14 + monocytes, although other myeloid lineage cells have also been implicated [50]. The process requires both M-CSF and RANKL, although the latter is considered the primary factor that differentiates pre-osteoclasts into osteoclasts, while M-CSF is responsible for their survival and proliferation [51]. RANKL functions through binding to its cognate receptor, RANK, which leads to the recruitment of TNF receptor–associated factors, such as TRAF6 (although multiple TRAF proteins are likely involved), which collectively activate multiple signaling pathways such as NF-κB, JNK, ERK, and NFATc1 [52–54]. The hallmark of identifying these cells in vitro and in vivo is positive staining for tartrate-resistant acid phosphatase (TRAP) and identification of multiple nuclei. These cells are typically large and form through the fusion of progenitor cells at the bone surface. TRAP staining results in a cell that is maroon colored through a hydrolytic reaction, which is easily identified in vitro [55]. Although it is widely viewed that CD14 + monocytes make up the majority of pre-osteoclast cells, recent evidence suggests that, in murine inflammatory arthritis, CD11c + dendritic cells (DC) are responsible for erosive disease, despite playing a reduced role in articular inflammation [56]. These conventional DCs were shown to be a major contributor to the pre-OC population in the joint and long bones. Further, cDCs (CD1c +) human cells can differentiate into osteoclasts [57].

Osteoclasts are the only known human cells which have the capability to degrade bone. These cells reside within the bone niche and promote bone turnover through a variety of mechanisms. First, the secretion of proton (H +) ions creates an acidic environment, which not only facilitates bone degradation but also enhances the survival and motility of osteoclasts [58]. Second, the release of key enzymes, known as cathepsin K, a cysteine protease, plays a crucial role in the degradation of bone in the organic phase. Cell–cell interaction between osteoclasts and local cells (osteoblasts, stromal cells) helps maintain bone homeostasis and survival of osteoclasts through the local production of M-CSF and RANKL. At the end of their life cycle, osteoclasts are thought to undergo apoptosis; however, there is increasing evidence in murine studies that osteoclast recycling (fission) may serve as an essential mechanism to regenerate osteoclasts [59]. However, it is not known if osteoclast fission occurs in humans nor if dysregulated osteoclast recycling is pathogenic in human disease.

## Neutrophils as First Responders in RA and PD-Associated Inflammation

One hallmark of inflammatory bone diseases is the massive infiltration of neutrophils in tissue. Neutrophil activation and neutrophil-mediated pathways can act as inflammatory triggers in inflammatory bone diseases [60–62] and activating resident immune cells [63]. Phagocytosis involves the internalization of pathogens, a process which ultimately leads to lysosomal-mediated processing [64]. Oxidative burst is facilitated by the enzyme MPO, which leads to the generation of oxygen-free radicals such as hydrogen peroxide [65]. These molecules can be released extracellularly or remain intracellularly in the phagolysosome [66].

Activated neutrophils are found in large numbers in the RA synovial fluid and pannus and in the oral cavity in PD [67–70]. In human PD, the number of neutrophils accumulated in the oral cavity correlates with the severity of periodontal tissue destruction [71]. Indeed, neutrophils have been implicated in the immunopathology of PD and RA [60, 61•, 72••, 73]. Furthermore, reduction of neutrophil infiltration in tissues has led to the protection of periodontal bone loss, supporting the role of neutrophils in mediating bone destruction [72••]. Extensive activity of neutrophils is harmful to host tissue because of the release of potent anti-microbial peptides and enzymes. For example, neutrophil activation through fibrin-CD11b binding can mediate inflammatory bone loss in experimental periodontitis [74•]; these findings support individuals harboring congenital deficiency in plasminogen that develops periodontitis [74•]. Neutrophil elastase, an enzyme that can be released through degranulation and during NET formation, can degrade extracellular matrix proteins including collagen and elastin as well as cartilage proteoglycans [75], although a direct effect on bone integrity has not been shown. Inhibition of NE in animal models of PD and RA ameliorates clinical manifestations and bone loss [76, 77]. Moreover, it has been reported that NE activity in gingival crevicular fluid from periodontitis patients correlates with disease severity [72••, 78]. One possible scenario is that NE can mediate proteolytic cleavage of osteoprotegerin (OPG), a negative regulator of osteoclasts, which acts as a decoy receptor for RANKL, thus leading to enhanced osteoclast formation and activity [76].

## NETs in Rheumatoid Arthritis and Periodontitis

The formation of NETs is viewed as a heterogeneous process, influenced predominantly by the stimuli and microenvironment of the neutrophils. However, there are several key events that occur which can skew neutrophils to undergo NET formation. First, a danger signal (immune complexes, crystals, cytokines, etc.) leads to the activation of the NADPH oxidase machinery promoting the generation of reactive oxygen species (ROS). The exact role of ROS in NET formation remains to be elucidated; however, in vitro studies of human neutrophils suggest that ROS is critical for the activation and nuclear translocation of serine proteases such as NE, to aid in the unwinding of densely packed chromatin, likely through the cleavage of histones [79]. Peptidylarginine deaminase (PAD)-4 is also activated, and this enzyme is involved in citrullination of histone proteins, converting arginine to citrulline with a resultant charge change that further influences DNA decondensation [80, 81]. Decondensed DNA mixes with cytosolic protein as NETs that are released to the extracellular space.

During NET formation, antigens such as citrullinated histones, DNA, and others are externalized. Excessive formation and/or impaired degradation of NETs in murine models of autoimmunity can trigger an aberrant adaptive immune response leading to autoantibody formation [10, 82]. NETs have been found in tissue specimens in RA and PD, and they have been associated with the pathogenesis of these conditions [73, 83]. NE is one of multiple enzymes that decorate NETs and is released upon NET formation. RA mouse models lacking NE display decreased inflammation, synovitis, and bone erosion [77]. This was supported by other mouse studies where pharmacologic inhibition of NE with the specific inhibitor sivelestat decreased cartilage damage and bone loss [75, 77]. Injection of NE into the joints induces acute inflammation and directly damages the cartilage in mouse models [75, 77]. NETs are present in synovial tissue and fluid of RA patients [73] and in oral cavity tissue in PD [72••, 83]. NETs can be triggered in vitro by bacteria that are known to play an important role in periodontitis such as *P. gingivalis* [84] and *Aggegatibacter actinomycetecomitans* [85, 86]. Furthermore, cytokines and immune complexes, among other sterile stimuli, can also trigger NETs in these tissues. In murine PD, fibrin accumulation from plasminogen deficiency and commensal microbiome interactions promotes pronounced neutrophilic infiltration. These neutrophils are activated by fibrinogen through C11b, a surface integrin, leading to the generation of ROS and the release of NETs. Intriguingly, in humans, polymorphisms in plasminogen are associated with severe PD and with colonization of *A. actinomycetemcomitans* [74•].

## NET Formation and Bone Turnover

In RA, degradation of NETs by serum-derived enzymes such as DNase I appears to be impaired compared to healthy controls [87]. Whether this impairment extends into the synovial joint is not clear; however, it is widely accepted that homeostatic processes that fail to degrade NETs may result in pronounced extracellular/bystander tissue damage, including the propagation of NET-mediated mechanisms that influence bone damage. In vitro, autoantibodies can induce NET formation, and this has been observed in numerous diseases, including systemic lupus erythematosus (SLE) and RA [73, 88]. Cigarette smoking has also been associated with NET formation in vitro and in vivo [89] and is clinically associated with risk for erosive RA [90] and the development of carbamylated RA autoantigens [91]. Thus, cigarette smoking and ACPA may be relevant stimuli of NET formation in RA, leading to further autoantibody production and early erosive disease. In RA synovium, elevated NETs can activate FLS and infiltrate T cells to produce RANKL [11•, 92]. In turn, RANKL instructs infiltrated monocytes to become osteoclasts using the canonical RANKL pathway [92]. Moreover, recent evidence from our group demonstrates that NETs directly induce osteoclast formation in vitro through the activation of TLR4 by histone H3 [92]. RANKL is not required for NET-mediated osteoclastogenesis, but this harnesses similar intracellular pathways, including Syk, Wnt, and PI3K activation. The TRAP + multinucleated cells generated by NETs are larger and less organized compared to RANKL/M-CSF-stimulated cells in vitro. Perhaps most interestingly, the process occurs in an extremely rapid manner; for example, in vitro generation of NET-osteoclasts occurs within 24 h, while RANKL/M-CSF-stimulated osteoclast formation typically takes 5–7 days, requiring supplementation of these cytokines on a frequent basis. These cells are functionally active and are apparent after intra-articular injection of heavily carbamylated NETs. This mechanism also seems to be operational in PD, since neutralization of histone H3 in an experimental model of periodontitis displayed significantly decreased bone loss (Kim). Genetic ablation or pharmacologic treatment against NE also displayed decreased bone loss in LIP [72••] (Fig. 2). These results demonstrate that NETs can influence osteoclast formation through district pathways, involving TLR activation and alternative signaling cascades. Understanding the role of NETs in bone metabolism provides insights into the mechanism of bone erosion in inflammatory diseases.

## Post-translational Modifications and Osteoclastogenesis

**Fig. 3:**
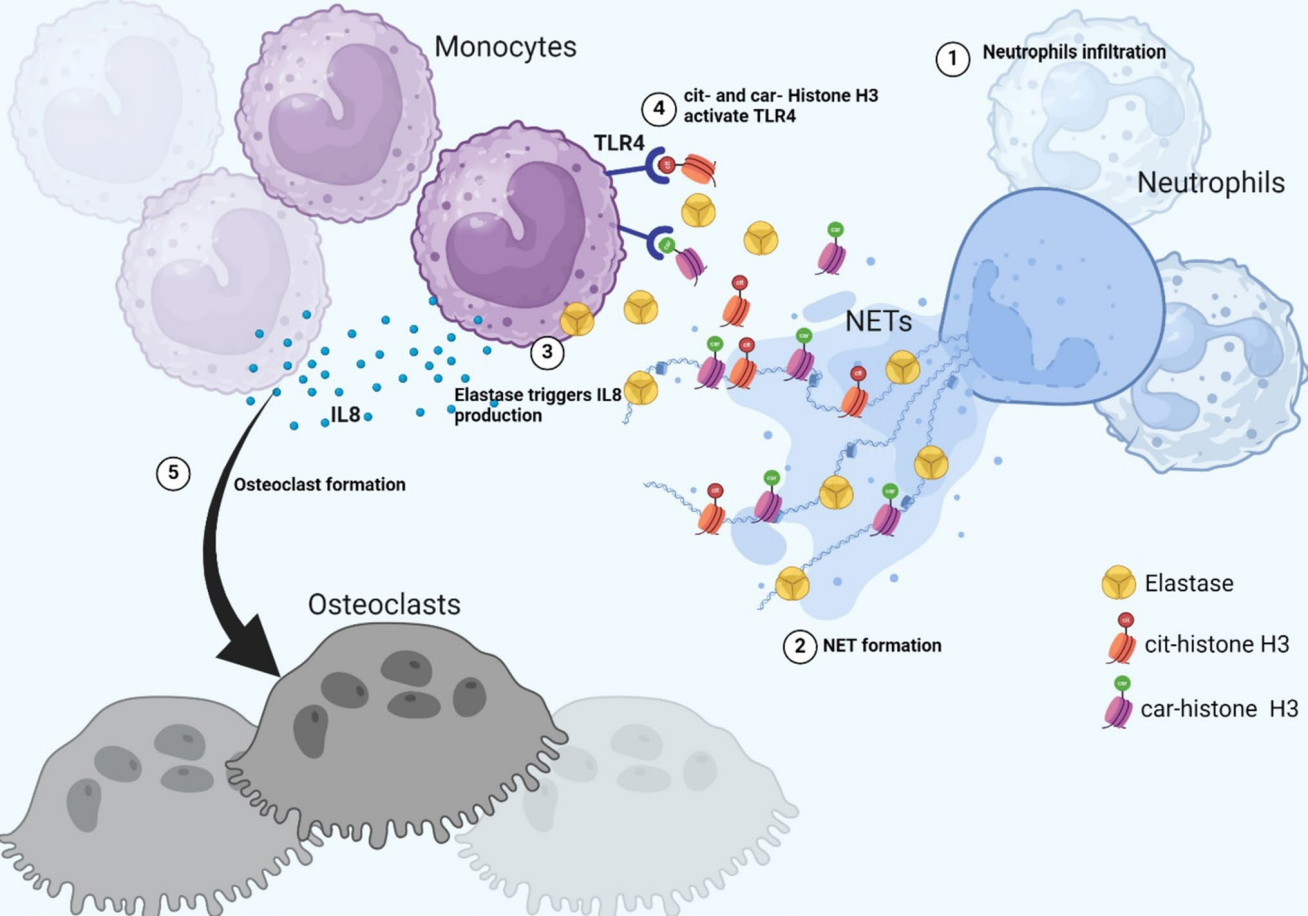
NETs and their modified protein cargo instruct monocytes to become osteoclasts. (1) Neutrophils infiltrate tissues (synovium or gingiva) and (2) release NETs containing citrullinated and carbamylated histones and elastase. (3) Neutrophil elastase stimulates IL-8 production by monocytes. (4) Citrullinated and/or carbamylated histones H3 activate TLR4 in monocytes, triggering an intracellular cascade that (5) leads to the formation of osteoclasts. Diagram generated using BioRender.com

Various PTMs, citrullination and carbamylation, have been linked to aberrant immune responses in RA and PD [93, 94]. Citrullination is a post-translational modification where arginine residues are converted into citrulline by PAD enzymes, while carbamylation is a nonenzymatic modification that occurs in lysine residues. Increased protein carbamylation and immune responses to carbamylated antigens have been described in RA and PD [94]. Citrullinated and carbamylated proteins are externalized during NET formation and can stimulate innate and adaptive responses leading to autoantibody formation [11•, 73]. Although over 80% of RA patients develop ACPAs, antibodies against carbamylated proteins (anti-CarP) are present in both RA and PD patients [95, 96]. ACPAs and anti-CarP antibodies have been associated with erosive bone disease in RA patients, suggesting their role in osteoclast formation and bone resorption. In fact, studies have shown that antibodies against citrullinated vimentin appear to bind to osteoclast surface enhancing osteoclast formation and increase bone resorption [97] (Fig. 1). Moreover, adoptive transfer of citrullinated vimentin antibodies in mice led to increased osteoclastogenesis accompanied with osteopenia due to increased TNF release [97]. Most of the studies have been focused on citrullinated vimentin antibodies in RA pathogenesis and bone loss, while research on anti-CarP has been limited. However, our lab identified that antibodies against carbamylated histones and other NET components are present in RA patients and correlate with X-ray bone erosion scores of the hands and feet. Anti-carbamylated histones H3 and H4 as well as LL37 antibodies potentiate osteoclast formation and resorption when compared to control IgG in vitro [11•, 98]. Furthermore, carbamylation of NETs significantly enhances their osteoclastogenesis potential, suggesting that PTMs play an important role in bone erosion. Moreover, proteases such as NE, also involved in NET-mediated osteoclastogenesis, are functionally enhanced through carbamylation, implicating a direct role for this PTM, and possibly citrullination, in facilitating osteoclast formation (Fig. 3). Elevated citrullinated and carbamylated proteins have also been reported in PD. Moreover, levels of citrullinated and carbamylated histone H3 are significantly elevated in serum and crevicular fluid of PD patients. These levels positively correlate with local bone destruction and overall disease severity [72••]. The intricate interplay of neutrophils, NETs, and PTM underscores the complexity of bone loss in inflammatory conditions. Understanding these mechanisms provides valuable insights for developing targeted therapies to mitigate bone erosion in RA and PD.

## Autoantibodies and Bone Loss

Given the known clinical association between the development of ACPA and erosive RA, there has been heightened interest in investigating the mechanisms by which autoantibodies drive bone loss. The most direct clinical evidence of this is the discovery that bone loss occurs in ACPA-positive individuals without RA, suggesting that bone loss may precede the clinical onset of inflammation [99]. Furthermore, ultrasonographic evidence of bone erosions in ACPA-positive individuals with arthralgias is predictive of progression into overt inflammatory arthritis [100], suggesting that it is an essential early stage in the transition from preclinical RA to clinical disease. Immune complexes signal predominantly through FcgR, which are expressed on numerous immune and stromal cells. For example, FcgR1, 2A, and 3A all signal through an ITAM (intrinsic immunoreceptor tyrosine–based activation motif) domain, leading to the phosphorylation of Syk. Similarly, osteoclasts express FcgRs, and through the common activation of Syk, this has a synergistic effect with RANKL stimulation, converging on the production of NFATc1, the master regulator of osteoclastogenesis. In vivo, ACPA can directly mediate bone loss in mice [101, 102], a process that is mediated, in part, through the enhanced formation of osteoclasts [103]. This interaction results in the release of key osteoclastogenic cytokines/chemokines; however, it is unlikely that the effect of ACPA on bone erosion in arthritis is solely due to the production of cytokines [104]. ACPA induces pre-osteoclast/monocyte populations to increase cytokines such as TNF, IL-1B, IL-6, and IL-8 through FcgR signaling [105], which further enhances osteoclastogenesis. IgG glycosylation can help facilitate the binding of the Fc portion of IgG to its FcgR, which is an important feature of ACPAs, specifically with respect to sialylation that likely plays a protective role in RA. Indeed, desialylated IgG immune complexes enhance osteoclastogenesis in vitro, compared to heavily sialylated IgG, which is also associated with reduced bone mass in patients with RA (102). Our group has reported that anti-carP, which is also clinically associated with erosive RA, can lead to enhanced osteoclast formation and function [11•]. It remains unknown if other antibodies, including rheumatoid factor, may be involved in osteoclast formation or bone erosion.

## Conclusion

The field of inflammatory bone disease has advanced substantially over the last decade, and it is abundantly clear that the processes underlying the imbalance of osteoclastic and osteoblastic activity are influenced strongly by a complex interplay between cytokines, immune cells, and local stromal cells. More recently, the role of neutrophils and the formation of NETs have been implicated in playing a substantial role in this bone turnover balance. NETs have direct and indirect effects on osteoclasts and clearly impact not only the activity of these bone degrading cells, but also their generation. Future research is needed to understand precisely how these pathways converge into the human phenotype, in both RA and periodontitis, so that therapeutics that disrupt this process can begin preclinical testing.

## Data Availability

No datasets were generated or analysed during the current study.
